# Photocatalytic Decomposition of Gaseous HCHO over Ag Modified TiO_2_ Nanosheets at Ambient Temperature

**DOI:** 10.3390/nano9030338

**Published:** 2019-03-02

**Authors:** Xueding Jiang, Weicheng Xu, Lian Yu

**Affiliations:** 1 School of Environmental and Chemical Engineering, Foshan University, Foshan 528000, China; jiangxueding@fosu.edu.cn (X.J.); weichengxu@fosu.edu.cn (W.X.); 2 College of Environment and Energy, South China University of Technology, Guangzhou 510006, China

**Keywords:** TiO_2_ nanocrystal, Exposed {001} facets, Ag nanoparticles, visible light, formaldehyde

## Abstract

Ag nanoparticles loaded onto TiO_2_ nanosheets with exposed {001} facets were synthesized by solvothermal hydrolysis and photoreduction deposition methods. The results suggested that Ag NPs were uniformly dispersed on the surface of anatase TiO_2_ NSs with a metallic state. The Raman scattering and visible light absorption performances of Ag/TiO_2_ NSs were enhanced by Ag NPs due to their surface plasmon resonance effect. Photocatalytic oxidation experiments for HCHO were carried out under visible light, and the enhanced photocatalytic activity of Ag/TiO_2_ NSs can be attributed to the synergistic effects of the following factors: (1) the {001} facets, which possessed higher surface energy, showed higher photocatalytic activity; (2) the Ag NPs, the increased oxygen vacancies, and O_2_ adsorption on {001} facets can trap photoelectrons, thus inhibiting the recombination of photoelectrons and holes; (3) the Ag NPs can extend the light response range of TiO_2_ into visible light. The in situ FTIR results showed that higher mineralization efficiency of HCHO was achieved on Ag/TiO_2_ NSs than on Ag/TiO_2_ NPs. Additionally, the mechanism for HCHO photocatalytic oxidation was also discussed.

## 1. Introduction

Formaldehyde (HCHO) is one of the most common volatile organic compounds (VOCs) in indoor air, which significantly decreases the indoor air quality and further influences human health [1]. As is well known, long-term exposure to HCHO brings serious health problems, such as pneumonia, headaches, and even lung cancer. Recently, many efforts have been made to eliminate indoor HCHO, such as adsorption [2,3], thermal catalytic oxidation [4], plasma technology [5], and photocatalytic oxidation [6,7]. However, because of their shortcomings: such as limited adsorption capacity, high reaction temperature, demands of high energy consumption, and low efficiency, etc., these methods are limited for the practical application [8].

As an environmental benign method, photocatalysis has been more frequently applied for VOCs removal, especially for indoor VOCs treatment [9]. HCHO can be degraded into innocuous final products (such as H_2_O and CO_2_) by photocatalytic oxidation (PCO) without significant energy input. The photocatalyst is one of the most important factors for photocatalytic reactions. Due to its low toxicity, strong stability, and excellent photocatalytic activity, TiO_2_ is widely used for the removal of organic compounds [10]. Since photocatalytic reactions mainly take place on the surface of the catalysts, the exposed crystal facets of the catalysts may remarkably affect the photocatalytic activity. Recently, TiO_2_ nanocrystals with different exposed crystal facets were synthesized, and the photocatalytic activity of these catalysts had been investigated. For anatase TiO_2_, experimental and theoretical studies have proved that {001} facets are more reactive than {101} facets [11,12], which is due to the abundant oxygen vacancies and higher surface energy [10]. It has been indicated that the photo-generated holes and electrons may have accumulated on the facets of {001} and {101}, and then they participated in photocatalytic oxidation and reduction reactions, respectively. These results indicated that the simultaneous exposure of {001} and {101} facets with appropriate proportions could improve the separation of photo-generated holes and electrons [11].

Nevertheless, due to the low quantum yields and ineffective visible light utilization, the application of TiO_2_ for photocatalytic degradation of VOCs is limited [13,14,15]. To solve these problems, some methods, such as metal ion or nonmetal ion doping and metal nanoparticles (NP) loading [13], have been used to improve the visible light photocatalytic activity. Very recently, noble metal modification has been proved to be an efficient approach for improving photocatalytic reactivity of TiO_2_; due to the surface plasmon resonance (SPR) effect, noble metal modification can improve the visible light photocatalytic activity [10,16]. The noble metals used include Au [17], Ru [18], Pd [19], Pt [20], and Ag [21]: among these noble metals, Ag was widely used for photocatalysis due to its low price and strong SPR absorption. The coupling of TiO_2_ with Ag can markedly facilitate the separation of photogenerated electrons and holes at the interface, thus improving the transfer efficiency of photocarriers. For example, after Ag clusters are loaded onto TiO_2_ nanosheets, the photocurrent can be increased 1.7 times [22]. 

Various methods, such as sol–gel process, impregnation, deposition–precipitation, and photodeposition, have been used to load Ag NPs onto TiO_2_ [13]. Among them, UV irradiation reduction combining deposition was a commonly used method, but the Ag NPs loaded onto TiO_2_ were amorphous [23]. The following calcination was needed to obtain crystalline Ag, and the heat treatment may result in the aggregation of Ag and TiO_2_ NPs, which was unfavorable for photocatalytic activity [23]. To overcome these disadvantages, film materials have been used to load Ag NPs [24]. Although Ag NPs have been successfully loaded onto TiO_2_ nanosheets [25], it is still difficult for Ag NPs to disperse well on TiO_2_ nanosheets.

As far as we know, the deposition of Ag NPs onto TiO_2_ nanosheets and investigation of the crystal facet dependent photocatalytic activity for HCHO degradation have not yet been reported in open publications. In this study, TiO_2_ with different exposed facets were synthesized via solvothermal hydrolysis, then Ag NPs were deposited onto TiO_2_ nanocrystals by photoreduction method, and the effect of the crystal facets on HCHO photocatalytic oxidation was examined. The mean size and the density of Ag NPs deposited on TiO_2_ could be tailored by controlling the reaction time and the concentration of Ag^+^. The HCHO oxidation experiments clearly revealed that the as-prepared Ag/TiO_2_ NSs with exposed {001} facets exhibited higher visible photocatalytic activities than Ag/TiO_2_ NPs with exposed {101} facets. The possible reasons for the enhanced photocatalytic activity over Ag/TiO_2_ NSs were discussed, and the reaction mechanism for the photocatalytic oxidation of HCHO was also proposed.

## 2. Experimental

### 2.1. Preparation of Ag/TiO_2_ NSs

TiO_2_ NSs were synthesized by solvothermal hydrolysis, which was reported by Yang et al. [12]. TiO_2_ NPs were synthesized under the same conditions, except for using deionized water instead of HF. Ag/TiO_2_ NSs/NPs were prepared by the photodeposition method. Typically, TiO_2_ was dispersed in methanol-water mixture (volume ratio of 1:1) under vigorous stirring, the obtained concentration of TiO_2_ was 1.0 g/L, and a certain amount of AgNO_3_ was added into the mixture. In nitrogen atmosphere, the mixture was exposed to a high pressure Hg lamp (400 W) for 4.0 h under stirring, and the obtained Ag/TiO_2_ catalysts were collected by filtration, washed with deionized water, dried under vacuum at 40 °C for 48 h, and then calcined at 250 °C for 2.0 h. The concentration of AgNO_3_ was 0.1 M, different volumes of AgNO_3_ solution were used to control the loading of Ag. The samples obtained were denoted as Ag/TiO_2_ NSs (NP) (x%), where x% indicated the Ag atom percentage of Ag/TiO_2_ catalysts. Ag NPs were prepared using the same method, but without the adding of TiO_2_. Ag/TiO_2_ NSs (5.0%) were used for all characterizations.

### 2.2. Characterization Methods

X-ray diffraction (XRD) patterns were obtained by a Philips X’Pert PRO X-ray diffraction instrument (Philips, Amsterdam, Holland). The morphologies were analyzed using a transmission electron microscopy (TEM), with a Tecnai G^2^ 20 S-TWIN microscope (FEI, Hillsboro, OR, USA). A Perkin-Elmer PHI 5000C ESCA system (Perkin-EImer, Waltham, MA, USA) was used to take X-ray photoelectron spectroscopy (XPS). N_2_ sorption isotherm was obtained using a Micromeritics TriStar II 3020 surface area analyzer (Micromeritics Instrument Corporation, Atlanta, GA, USA). Photoluminescence spectroscopy (PLS) and UV–vis diffuse reflectance spectra (UV–vis DRS) were collected on a Varian Cary-Eclipse 500 and MC-2530 (Varian, Palo Alto, CA, USA), respectively. Raman spectra were taken by a Via Reflex Raman spectrometer (Renishaw plc, London, UK), and the excitation wavelength was 325 nm. A Nicolet 6700 FT-IR spectrometer (Thermo Fisher Scientific, Waltham, MA, USA) was used to analyze the in situ diffuse reflectance infrared Fourier transform (DRIFT) spectra. The loading of Ag nanoparticles in the catalysts were determined by an inductive coupled plasma emission spectrometer (VISTAMPX-ICP) (Varian, Palo Alto, CA, USA).

### 2.3. Degradation Experiment

**Figure 1:**
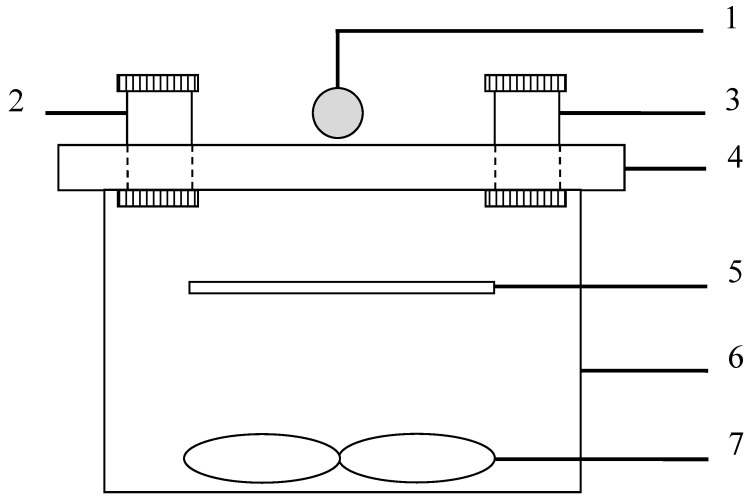
Experimental facility for photocatalytic degradation of HCHO: (**1**) light source (a 500 W xenon lamp), (**2**) humidity controller, (**3**) sampling/injection port, (**4**) quartz cover, (**5**) glass plate with photocatalyst, (**6**) stainless steel container, (**7**) electric fans.

A self-made airtight reactor with a quartz cover was applied to carry out the experiments, its size was 500 mm (L) × 300 mm (W) × 300 mm (H) (Figure 1). In order to eliminate the UV light, a glass cut filter (λ > 400 nm; the output wavelength, 400–780 nm) was equipped to the xenon lamp. The light incident intensity was ca. 10.0 ± 0.5 mW/cm^2^ at the surface sites of the photocatalysts. Two electric fans were used to circulate the mixture of HCHO and air. The initial concentration of HCHO was set at 0.50 ppm (photocatalyst dosage, 0.20 g). During experiments, sampling was made through the septum of the sampling/injection port at certain time intervals. The experiments were carried out at room temperature (25 ± 1 °C). The relative humidity (RH) for the reactant gas was measured online by a dew point hygrometer (635-2, Testo, Schwarzwald, Germany). The photocatalytic activity was assessed by the photocatalytic degradation efficiency of HCHO, and the concentration of HCHO was measured by the acetylacetone spectrophotometric method [26]. The concentration of CO_2_ was analyzed by on-line gas chromatograph, which was equipped with FID (Flame Ionization Detector) detectors: a converter with nickel catalyst was placed before the FID detector and it was used for converting CO_2_ into methane in the presence of H_2_.

## 3. Results and Discussion

### 3.1. Structural Characteristics

**Figure 2:**
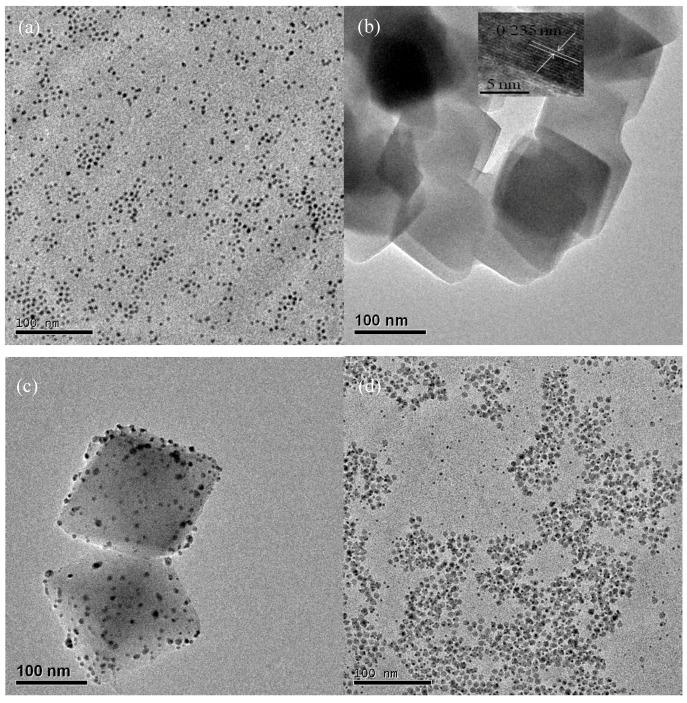
TEM images of Ag NPs (**a**), TiO_2_ NSs (**b**), Ag/TiO_2_ NSs (**c**), and Ag/TiO_2_ NPs (**d**).

Figure 2a displayed the structural characterization of the as-prepared Ag NPs. The Ag NP was spherical with an average diameter of 5.0~8.0 nm. As can be seen from Figure 2c,d, Ag NPs with sizes of 2~4 nm were uniformly dispersed on the surface of Ag/TiO_2_ NSs and Ag/TiO_2_ NPs. It can be seen that the diameters of Ag NPs in Ag/TiO_2_ NSs and Ag/TiO_2_ NPs were smaller than that of pure Ag NPs, which is due to the support effect of TiO_2_ NSs and NPs. Figure 2b,c revealed that TiO_2_ NSs and Ag/TiO_2_ NSs were both present in nanosheets, with an average length of 100–150 nm. By comparing Figure 2b,c, it can be seen that the deposition of Ag NPs did not obviously influence the morphologies of TiO_2_ nanosheets. TiO_2_ NPs in Ag/TiO_2_ NPs (Figure 2d) displayed spherical morphology, with an average size of around 10 nm. The HRTEM (High Resolution Transmission Electron Microscopy) image (inset of Figure 2b) of the side face of TiO_2_ NSs directly showed that the lattice spacing parallel to the top and bottom facets was 0.235 nm, which can be ascribed to the (001) plane of anatase TiO_2_, proving that most Ag NPs were deposited on the {001} facets of TiO_2_.

**Figure 3:**
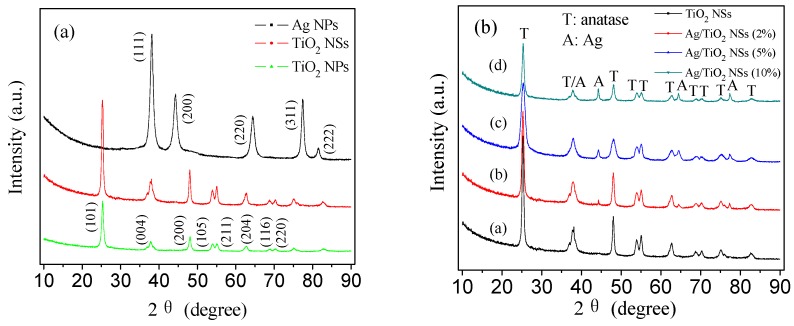
XRD patterns of pure catalysts (**a**) and Ag/TiO_2_ NSs catalysts (**b**).

Figure 3a showed the XRD patterns of Ag NPs, Ag/TiO_2_ NSs (5%), and Ag/TiO_2_ NPs (5%). The peaks at 2θ = 38.1°, 44.3°, 64.4°, 77.4°, and 81.5° can be assigned to the diffraction for (111), (200), (220), (311), and (222) lattice planes of the cubic Ag (JCPDS (Joint Committee on Powder Diffraction Standards) 87–0597), respectively. No peak belonging to silver oxide has been observed, indicating high purity metallic silver [27]. For TiO_2_ NSs and TiO_2_ NPs, the diffraction peaks at 2θ = 25.3°, 37.8°, 48.0°, 53.9°, 55.1°, and 62.7° can be ascribed to (101), (004), (200), (105), (211), and (204) crystal planes of anatase TiO_2_ (JCPDS 21–1272), respectively, indicating their pure anatase TiO_2_ phase. However, the relative intensity of peaks for TiO_2_ NSs were higher than those for TiO_2_ NPs, indicating that TiO_2_ NSs showed higher crystallinity of anatase than TiO_2_ NPs. Figure 3b showed the XRD patterns of TiO_2_ NSs and Ag/TiO_2_ NSs with different contents of Ag. As can be seen, TiO_2_ in TiO_2_ NSs and Ag/TiO_2_ NSs all exhibited the anatase structure, and the characteristic 2θ values were at 25.3°, 37.8°, 48.0°, 53.9°, 55.1°, and 62.7°, respectively (JCPDS 21−1272). The peaks of Ag/TiO_2_ NSs that indexed to anatase TiO_2_ were the same as TiO_2_ NSs, indicating that the deposition of Ag NPs did not change the lattice structure of anatase TiO_2_. Moreover, no diffraction peaks ascribed to Ag NPs were found in Ag/TiO_2_ NSs (2%), which can be due to the low content of Ag NPs and their high dispersion in Ag/TiO_2_ NSs. For Ag/TiO_2_ NSs (5%) and Ag/TiO_2_ NSs (10%), besides the diffraction peaks for anatase TiO_2_, the other diffraction peaks can be ascribed to metallic Ag [27].

**Figure 4:**
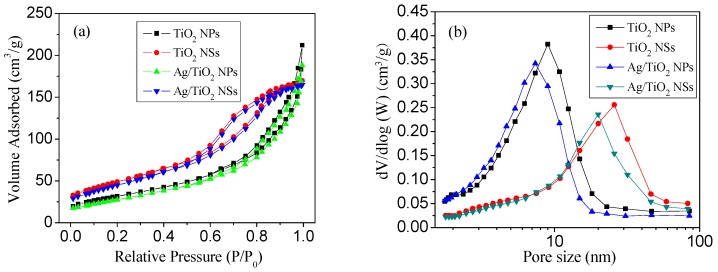
N_2_ adsorption-desorption isotherms (**a**) and pore size distribution curves (**b**) of different catalysts.

As can be seen from N_2_ adsorption−desorption isotherms (Figure 4), TiO_2_ NSs and Ag/TiO_2_ NSs showed typical type IV N_2_ adsorption-desorption isotherms, with H_3_ hysteresis loops at relative pressure of 0.5–1.0, which can be due to the aggregates of plate-like particles which formed slit-like pores [28]. TiO_2_ NPs and Ag/TiO_2_ NPs displayed type IV isotherm, with a H_2_ hysteresis loop, which was associated with the presence of pores due to the agglomerates of nanoparticles [28]. It was known that the reactant and product molecules could be efficiently transported through the porous structures [28], therefore, the presence of porous structures could improve the photocatalytic activity of Ag/TiO_2_ NSs. The corresponding pore size distribution curves (Figure 4b) showed that the average pore diameters of TiO_2_ NPs, TiO_2_ NSs, Ag/TiO_2_ NPs, and Ag/TiO_2_ NSs were 8.99 nm, 25.83 nm, 7.37 nm, and 20.04 nm, respectively. Based on the N_2_ adsorption-desorption isotherms, the S_BET_ of TiO_2_ NPs, TiO_2_ NSs, Ag/TiO_2_ NPs, and Ag/TiO_2_ NSs were 132.17, 98.45, 124.26, and 94.52 m^2^/g, respectively. The pore size and surface area remained almost unchanged after the loading of Ag, indicating that the deposition of Ag NPs did not evidently influence the pore size and surface area of TiO_2_. It can be seen that the surface area of Ag/TiO_2_ NSs was smaller than that of Ag/TiO_2_ NPs, indicating that the surface area was not the main factors for the high activity of Ag/TiO_2_ NSs.

**Figure 5:**
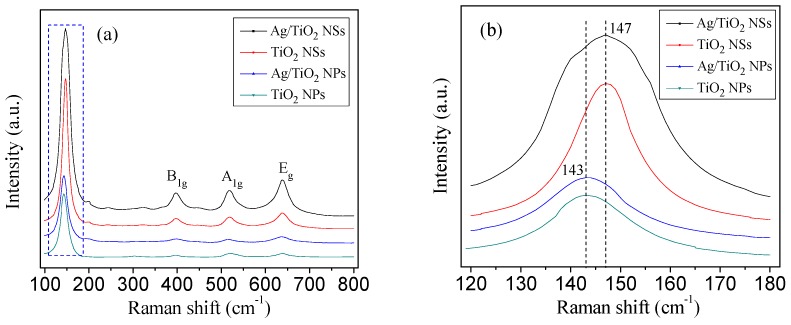
Raman spectra of different samples (**a**) and the magnified Raman spectra from 120 to 180 cm^−1^ (**b**).

As shown in Figure 5a, all the samples were of the similar peaks, locating at 144 (Eg), 197, 394 (B1g), 514 (A1g), and 636 cm^−1^ (Eg), indicating the anatase phase of the samples. It also indicated that the deposition of Ag NPs did not affect the nanostructure of anatase TiO_2_. Furthermore, no silver oxide peak can be detected in the Ag/TiO_2_ catalysts, indicating that the Ag NPs in the catalysts were high-purity metal silver. As can be seen from the magnified Raman spectra from 120 to 180 cm^−1^ (Figure 5b), when comparing the peaks of TiO_2_ NSs and Ag/TiO_2_ NSs with that of TiO_2_ NPs and Ag/TiO_2_ NPs, a positive shift by about 4.0 cm^−1^ can be observed, indicating the increase of surface oxygen vacancies on the {001} facets [10]. Ti atom in {101} facets is coordinated with either 5 or 6 oxygen atoms (with a probability of around 50%), but in {001} facets, Ti atom is coordinated with 5 oxygen atoms [29]. It can be expected that the {001} facets may have more surface oxygen vacancies than {101} facets, and more surface oxygen vacancies are favorable for photocatalytic activity.

**Figure 6:**
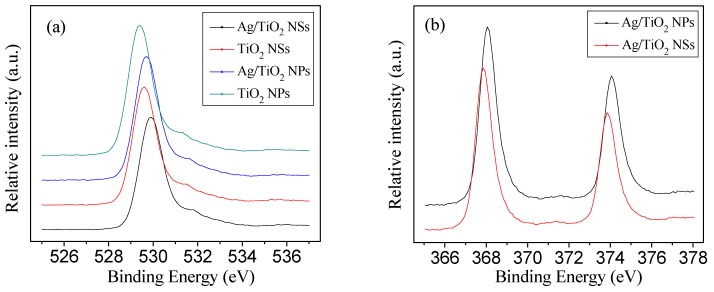
XPS spectra of O 1s for four catalysts (**a**) and Ag 3d for Ag/TiO_2_ (NSs and NPs) (**b**).

As shown in Figure 6a, as for O 1s XPS spectrum, the peak at 529.7 eV can be attributed to the O−Ti bond on the surface of TiO_2_ loaded by Ag NPs. Compared with TiO_2_ NSs and TiO_2_ NPs, the peaks of O 1s for Ag/TiO_2_ NSs and Ag/TiO_2_ NPs shifted towards higher binding energy (from 529.6 to 529.9 eV and from 529.4 to 529.7 eV, respectively), which suggested that due to the electron trapping ability of Ag NPs, less electron density remained at the oxygen atom in Ag/TiO_2_ than in pure TiO_2_. As can be seen in Figure 6b, the binding energy of Ag 3d_5/2_ and Ag 3d_3/2_ for Ag/TiO_2_ NSs was 367.9 and 373.9 eV, respectively, and the related binding energy for Ag/TiO_2_ NPs was 368.1 and 374.1 eV, respectively. The spin energy separation were all 6.0 eV, which indicated that the silver species existing in TiO_2_ were metallic Ag^0^ [30]. The binding energy of O 1s in TiO_2_ NSs and Ag/TiO_2_ NSs shifted positively by around 0.20 eV with regards to that of TiO_2_ NPs and Ag/TiO_2_ NPs, while the binding energy of Ag 3d_5/2_ and 3d_3/2_ in Ag/TiO_2_ NSs shifted negatively by around 0.20 eV with regards to that of Ag/TiO_2_ NPs, which also indicated the enhanced amount of surface oxygen vacancies for Ag/TiO_2_ NSs [10]. Due to surface oxygen vacancies, more O_2_ molecules may have adsorbed onto the surface of Ag/TiO_2_ NSs, and the O_2_ molecules can react with photogenerated electrons to form ^•^O_2_^−^, which inhibited the recombination of photogenerated electrons and holes [30].

**Figure 7:**
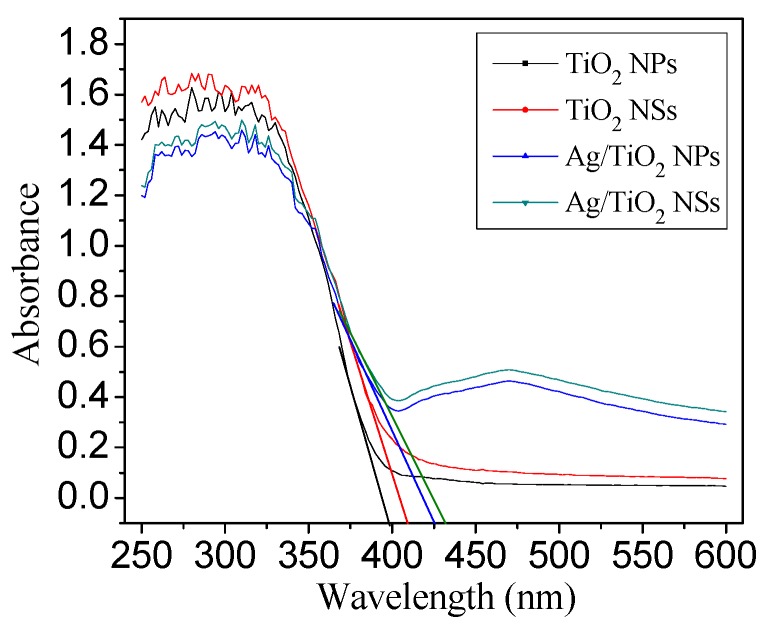
UV–vis DRS spectra of different catalysts.

From Figure 7, in contrast to pure anatase TiO_2_, an additional absorption band centered at ca. 560 nm (visible light range) can be observed in both Ag/TiO_2_ NSs and Ag/TiO_2_ NPs, which was the surface plasmon resonance (SPR) absorption band for metallic Ag NPs [31]. This would contribute to the enhanced visible light photocatalytic activity [11]. As can be seen from the UV–vis DRS spectra from 380 to 600 nm, the absorption capability for visible light varied in the order of Ag/TiO_2_ NSs > Ag/TiO_2_ NPs > TiO_2_ NSs > TiO_2_ NPs. TiO_2_ NSs and Ag/TiO_2_ NSs showed stronger absorption capability than the corresponding TiO_2_ NPs and Ag/TiO_2_ NPs, which was attributed to the different surface energy; the {001} facets showed a more decreased energy band gap (3.18 eV) than the {101} facets (3.22 eV) [10]. The increased absorption in visible light region and the decreased band gap of TiO_2_ made Ag/TiO_2_ NSs an efficient visible light photocatalyst [25].

**Figure 8:**
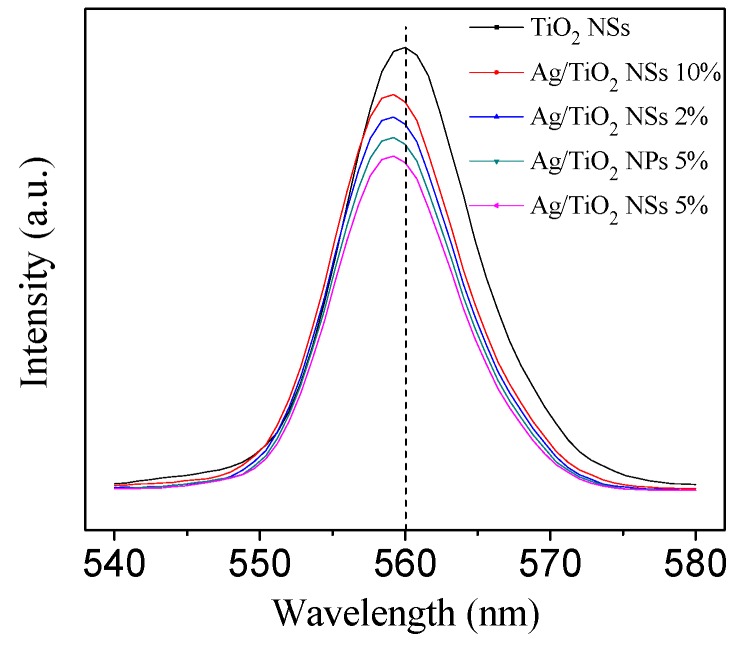
PL spectra of different catalysts.

Figure 8 revealed that the intensity of PL emission around 560 nm decreased in the order of TiO_2_ NSs > Ag/TiO_2_ NSs (10%) > Ag/TiO_2_ NSs (2%) > Ag/TiO_2_ NPs (5%) > Ag/TiO_2_ NSs (5%), indicating the decrease of the recombination rate for photoelectrons and holes [10]. Ag/TiO_2_ NSs (5%) exhibited a lower recombination rate for photoelectrons and holes than Ag/TiO_2_ NPs (5%). There were more surface oxygen vacancies on the surface of Ag/TiO_2_ NSs (5%): these oxygen vacancies could capture photoelectrons, and that inhibited their recombination with holes [29]. Ag/TiO_2_ NSs (5%) and Ag/TiO_2_ NPs (5%) exhibited lower photoelectrons and holes recombination rate, as compared to TiO_2_ NSs and NPs, which was due to Ag NPs, which can function as electron sinks to trap photoelectrons, and thus reduced their recombination with holes. Meanwhile, Figure 8 revealed that the intensity of emission peak decreased with the increase of the Ag loading up to 5.0% for Ag/TiO_2_ NSs, but when the Ag loading exceeded 5.0%, the photoelectron-hole recombination rate increased with the increase of Ag loading, which is due to the increase in the size of Ag nanoparticles and the poor interaction between Ag and TiO_2_ in the catalysts.

### 3.2. Photocatalytic Performance

**Figure 9:**
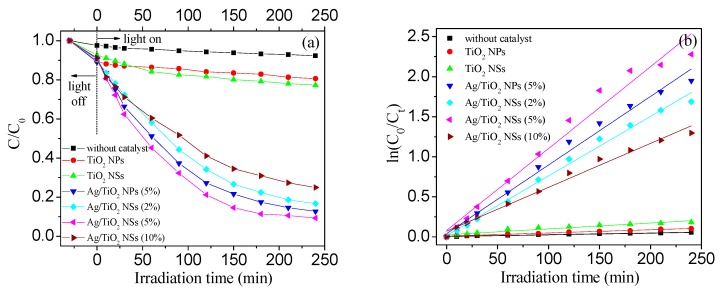
(**a**) Time-dependent degradation efficiency of gaseous HCHO by different catalysts under visible light; (**b**) the related degradation kinetics. Conditions: RH=45%, C_0_ (HCHO) = 0.5 ppm.

Before irradiation, a decrease of the HCHO concentration can be found (Figure 9a), which is due to the sorption of HCHO on the photocatalysts and on the inner wall of the reactor. Figure 9a showed that the photocatalytic activity ranked in the order of Ag/TiO_2_ NSs (5%) > Ag/TiO_2_ NPs (5%) > Ag/TiO_2_ NSs (2%) > Ag/TiO_2_ NSs (10%) > TiO_2_ NSs > TiO_2_ NPs. Among the catalysts, Ag/TiO_2_ NSs (5%) exhibited the highest oxidative activity for HCHO (91.3% of removal efficiency for 240 min irradiation). Ag/TiO_2_ NSs and Ag/TiO_2_ NPs exhibited higher activity than the related TiO_2_ NSs and TiO_2_ NPs, which can be ascribed to the synergistic effects between Ag and TiO_2_, resulting from the reduced hole-electron recombination and the increased visible absorption [32] (Figure 7). Meanwhile, TiO_2_ NSs and Ag/TiO_2_ NSs (5%) showed higher activity than the related TiO_2_ NPs and Ag/TiO_2_ NPs (5%): since all the catalysts had almost the same surface area, the enhanced activity should be associated with the other chemical and physical factors, such as the light-harvesting, the ratio of exposed {001} facets, the number of surface oxygen vacancies, and the crystal size [10]. Based on the above results, two conclusions can be drawn: (1) TiO_2_ NSs with exposed {001} facets showed higher activity than TiO_2_ NPs with exposed {101} facets, indicating a strong crystal facet-dependent activity; and (2) Ag NPs-loaded TiO_2_ nanocrystals exhibited higher photocatalytic activity than pure TiO_2_ nanocrystals.

In order to analyze the kinetic process of the photocatalytic reactions, the experimental results from Figure 9a were fitted by the pseudo first-order model, which can be expressed as Equations (1) and (2):

(1) $$\ln(C_{0}/C_{t})=kt,$$

(2) $$r_{0}=-\frac{dC}{dt}=kC_{0},$$

Therein, *k* is the rate constant of apparent reaction, *C*_0_ is the initial concentration of HCHO, *C*_t_ is the concentration of HCHO at time *t*, *r*_0_ is the initial photocatalytic degradation rate, and R^2^ is the correlation coefficients. The results can be seen in Figure 9b and Table 1. The kinetic curves were corresponding to the practical situation, suggesting that the HCHO degradation processes depended linearly on the HCHO concentration, which was common in low concentration gas-phase reactions [33]. It was found that the k for Ag/TiO_2_ NSs (5%) (*k* = 0.01022/min) was 14.0 times higher than that of pure TiO_2_ NSs (*k* = 0.00073/min) and 1.2 times higher than that of Ag/TiO_2_ NPs (5%) (*k* = 0.00856/min). These results indicated that Ag/TiO_2_ NSs (5%) showed the highest photocatalytic activities.

**Figure 10:**
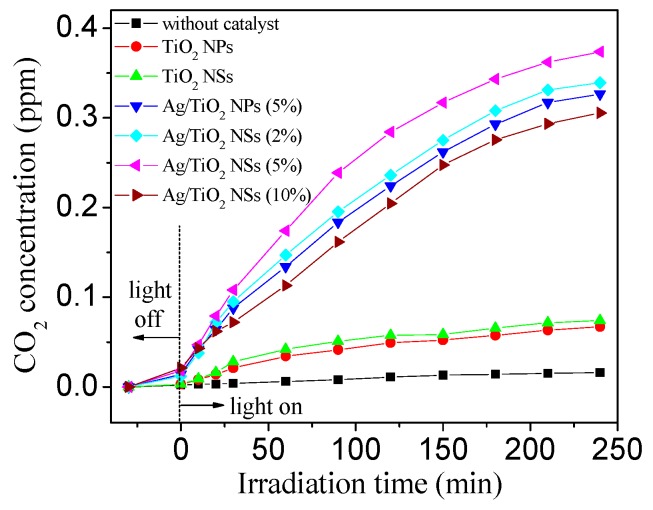
The changes of CO_2_ concentration during HCHO photocatalytic degradation by different catalysts.

The changes of CO_2_ concentration during HCHO photocatalytic degradation can be seen in Figure 10. Under dark conditions, a small amount of CO_2_ corresponding to HCHO oxidation was detected, and the mineralization of HCHO occurred at a low level. This indicated that HCHO was removed mainly via adsorption by the catalysts under dark conditions. After the visible light was on, the HCHO concentration quickly decreased (Figure 9a), accompanied by CO_2_ concentration increase (Figure 10), which can be attributed to the photocatalytic oxidation of HCHO. As can be seen from Figure 10, CO_2_ concentration followed the order: Ag/TiO_2_ NSs (5%) > Ag/TiO_2_ NPs (5%) > Ag/TiO_2_ NSs (2%) > Ag/TiO_2_ NSs (10%) > TiO_2_ NSs > TiO_2_ NPs. For pure TiO_2_ NPs and TiO_2_ NSs, less CO_2_ was detected. The loading of a small amount of Ag NPs significantly enhanced the photocatalytic activity of Ag/TiO_2_ for the mineralization of HCHO. Ag/TiO_2_ NSs (5%) showed the highest mineralization efficiency for HCHO, and 0.37 ppm CO_2_ can be detected at the time of 240 min: as the initial concentration of HCHO was 0.5 ppm, the corresponding mineralization efficiency was 74.00%. With an increase or decrease of the Ag loading, the mineralization efficiency of the catalysts for HCHO decreased, which was consistent with the HCHO degradation experiments (Figure 9).

**Figure 11:**
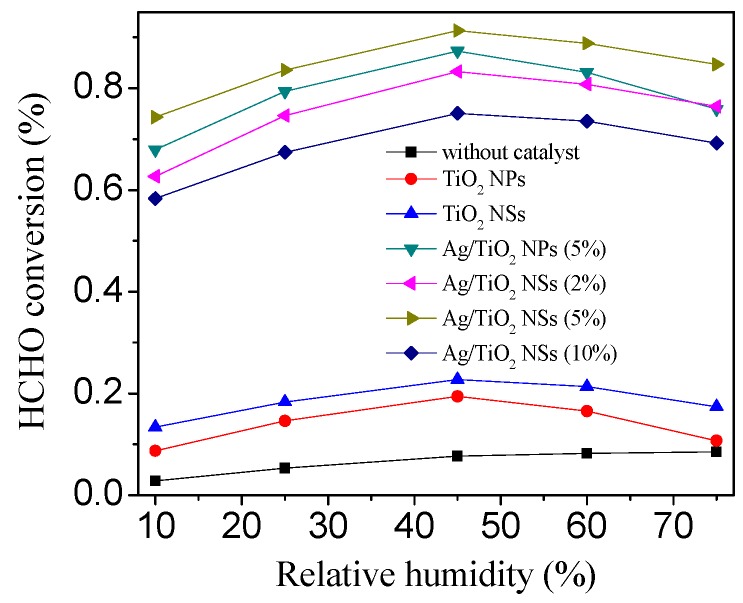
Effect of relative humidity on the removal efficiency of HCHO by different catalysts under visible light irradiation for 240 min.

Figure 11 showed the HCHO removal efficiency by different catalysts when the relative humidity was adjusted from 10% to 75%. It can be seen that the optimal RH was 45%, both TiO_2_ and Ag/TiO_2_ depended weakly on the RH in the experimental range, but RH may play an important role for HCHO degradation. The high RH was beneficial to generate ^•^OH in the gas phase [32]. Thus, the presence of water vapor can enhance the photocatalytic activity. Nevertheless, if the RH was too high, the photocatalytic degradation of HCHO on the surface of Ag/TiO_2_ would be inhibited because of the competitive adsorption of HCHO and H_2_O [32]. For these two opposite coexisting factors, the total HCHO removal efficiency on Ag/TiO_2_ slightly varied as the RH increased from 10% to 75%. With the increasing of the RH, the HCHO decomposition increased gradually. When the RH exceeded 45%, the HCHO removal efficiency decreased gradually with the increasing of RH. Nevertheless, even under the RH of 10% and 75%, Ag/TiO_2_ NSs (5%) still showed excellent photocatalytic activity, indicating that the activity of Ag/TiO_2_ NSs (5%) was sufficiently high.

**Figure 12:**
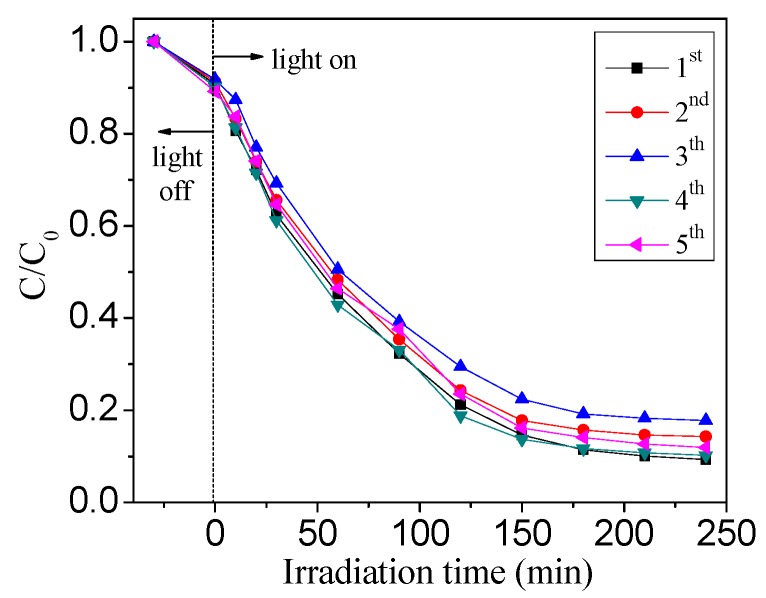
Five cycles of photocatalytic degradation of HCHO over Ag/TiO_2_ NSs (5%).

The reusability of Ag/TiO_2_ NSs for photocatalytic degradation of HCHO was evaluated, as shown in Figure 12. At the end of each cycle, Ag/TiO_2_ NSs were taken out and then placed indoor for 12 h. After five cycled runs for HCHO photocatalytic degradation, the photocatalytic activity of Ag/TiO_2_ NSs did not exhibit great decrease, and the removal efficiency still maintained over 85%, suggesting the excellent reusability of Ag/TiO_2_ NSs. 

### 3.3. Mechanism of HCHO Photocatalytic Oxidation on Ag/TiO_2_ NSs

**Figure 13:**
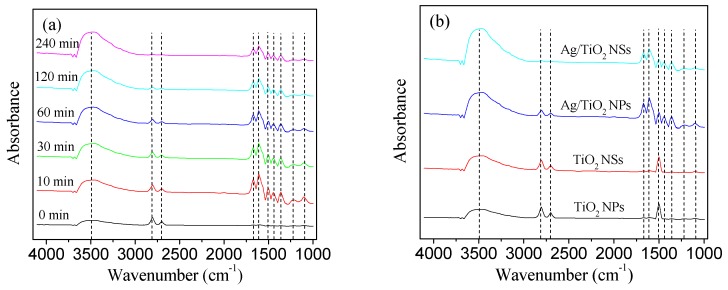
(**a**) Dynamic changes in the in situ DRIFT spectra of HCHO photocatalytic oxidation under visible light; (**b**) the in situ DRIFT spectra of different catalysts for photocatalytic oxidation of HCHO at 120 min.

The reaction mechanisms of HCHO photocatalytic oxidation were investigated by in situ diffuse reflectance infrared Fourier-transform (DRIFT) spectra (Figure 13) at room temperature. As can be seen from Figure 13a, two small bands (around 3500 cm^−1^ for ν(OH) and 1670 cm^−1^ for δ(H–O–H)) can be observed after Ag/TiO_2_ NSs were exposed to HCHO/O_2_ for 30 min in the dark (0 min in Figure 13a), suggesting the existing of chemisorbed H_2_O on Ag/TiO_2_ NSs surface. The intensities of the two bands gradually increased with the increasing reaction time, because HCHO was further oxidized and more H_2_O was generated. After 10 min visible light irradiation, dioxymethylene species (1500 cm^−1^ for δ(CH2) and 1100 cm^−1^ for ν(CO)) and formate species (2830, 2807, and 2703 cm^−1^ for ν(CH); 1610, and 1435 cm^−1^ for ν_as_(COO); 1360 and 1320 cm^−1^ for ν_s_(COO); 1215 cm^−1^ for ν(CO)) can be observed in the in situ DRIFT spectra [34]. With the increase of reaction time, the intensities of the bands for dioxymethylene and formate species decreased, which is attributed to the oxidation of dioxymethylene and formate species.

It can be clearly observed (Figure 13b) that the absorption peaks which were attributed to H_2_O and formate species can be detected for Ag/TiO_2_ NSs and Ag/TiO_2_ NPs. As for TiO_2_ NSs and TiO_2_ NPs, the intensities of peaks attributed to H_2_O and formate species were weaker than those of Ag/TiO_2_ NSs and Ag/TiO_2_ NPs. These results suggested that HCHO cannot be efficiently oxidized by TiO_2_ NSs and TiO_2_ NPs under visible light. Comparing the intensities of peaks for the formate species, they cannot be detected in the spectra of pure TiO_2_ NPs and TiO_2_ NSs; they can be observed in the spectra of Ag/TiO_2_ NPs and Ag/TiO_2_ NSs. It can be seen that the intensities of peaks of the formate species for Ag/TiO_2_ NSs was lower than that of Ag/TiO_2_ NPs, indicating lower accumulation of intermediates on Ag/TiO_2_ NS surface: the rapid desorption or decomposition of intermediates would contribute to the high activity and stability of Ag/TiO_2_ NSs.

The enhanced photocatalytic activity of Ag/TiO_2_ NSs can be attributed to the following factors: (1) Ag NPs may function as electron sinks, which can trap the photoelectrons and reduce their recombination with holes; (2) the Ag/TiO_2_ NSs allow the adsorption of visible light; (3) there was a certain correlation between photocatalytic activity and surface energy, as it can be seen that Ag/TiO_2_ NSs exposed with {001} facets showed higher photocatalytic activity than Ag/TiO_2_ NPs exposed with {101} facets; (4) the Ag NPs were uniformly deposited on the surface of TiO_2_ NSs, which eliminated the aggregation of TiO_2_ NSs obtained under the same conditions.

**Figure 14:**
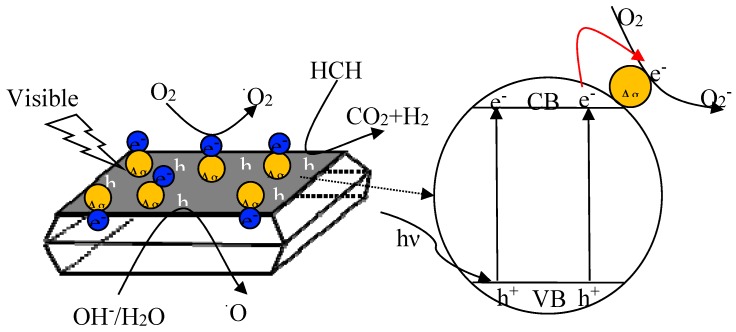
Schematic diagram illustrating the photocatalytic oxidation process of HCHO over Ag/TiO_2_ NSs under visible light irradiation.

On the basis of the above results, the reaction mechanism for the photocatalytic degradation of HCHO over Ag/TiO_2_ NSs was proposed (Figure 14). Firstly, HCHO was adsorbed onto the surface of Ag/TiO_2_ NSs [35]. Secondly, under visible light irradiation, the photoelectrons were transformed from the valence band to conduction band of TiO_2_ NSs, leaving holes at the valence band. Thirdly, the generated electrons and holes reacted with O_2_ or H_2_O molecules to form oxy radicals, including superoxide radicals (^•^O_2_^−^) and hydroxyl radicals (^•^OH) [35]. Finally, the produced ^•^O_2_^−^ and ^•^OH could participate in HCHO oxidation, HCHO was decomposed into formate species, and then H_2_O and CO_2_ [35]. The mechanism for HCHO photocatalytic oxidation by Ag/TiO_2_ can be described by the following reactions:

(3) $$Ag/TiO_{2}+h\upsilon \to e^{-}+h^{+},$$

(4) $$e^{-}+O_{2}\to {}^{•}O_{2}^{-},$$

(5) $$h^{+}+OH^{-}\to {}^{•}OH,$$

(6) $${}^{•}OH+HCHO\to H_{2}O+{}^{•}CHO,$$

(7) $${}^{•}CHO+{}^{•}O_{2}^{-}+H^{+}\to H_{2}CO_{3}\to H_{2}O+CO_{2},$$

## 4. Conclusions

In summary, this study developed a facile method to load Ag NPs onto TiO_2_ NSs with exposed {001} facets. Ag/TiO_2_ NSs exhibited high activity for HCHO photocatalytic oxidation under visible light. Compared with TiO_2_ NPs, TiO_2_ NSs, and Ag/TiO_2_ NPs, Ag/TiO_2_ NSs (5%) exhibited the highest photocatalytic activity. The enhancement of photocatalytic activity of Ag/TiO_2_ NSs can be due to the synergetic promoting effects of Ag NPs and TiO_2_ NSs, such as the greater ratio of {001} facet exposure, more surface oxygen vacancies, and stronger visible light harvesting. In situ DRIFT studies indicated that HCHO was first chemisorbed and reacted with the ^•^O_2_^−^ and ^•^OH to form formate species, and then H_2_O and CO_2_. Meanwhile, Ag/TiO_2_ NSs displayed excellent durability, indicating Ag/TiO_2_ NS is a promising catalyst for indoor HCHO purification.

## Figures and Tables

**Table 1:** The related parameters obtained from kinetic study.

Sample	*C*_0_ (mg/L)	*k* (min^−1^)	R^2^	*r*_0_ (mg/(L·min))
blank	0.488	0.00022	0.9883	0.00011
TiO_2_ NPs	0.447	0.00040	0.9917	0.00018
TiO_2_ NSs	0.464	0.00073	0.9639	0.00034
Ag/TiO_2_ NPs (5%)	0.446	0.00856	0.9945	0.00382
Ag/TiO_2_ NSs (2%)	0.452	0.00743	0.9964	0.00335
Ag/TiO_2_ NSs (5%)	0.454	0.01022	0.9884	0.00464
Ag/TiO_2_ NSs (10%)	0.456	0.00548	0.9943	0.00250
